# Serum dithiothreitol-oxidizing capacity (DOC) is a promising biomarker for excluding significant liver fibrosis: a proof-of-concept study

**DOI:** 10.1186/s12916-024-03502-z

**Published:** 2024-07-02

**Authors:** Lumin Yang, Yafei Zhang, Xiaodan Hong, Ke Zhang, Bingyan Liu, Peixin Zhang, Qianqian Tang, Jian Yu, Xiao-Zhi Jin, Xin-Zhe Jin, Ni Zhang, Giovanni Targher, Christopher D. Byrne, Zhenhua Zhang, Ming-Hua Zheng, Jinsong Zhang

**Affiliations:** 1 State Key Laboratory of Tea Plant Biology and Utilization, School of Tea and Food Science, Anhui Agricultural University, No. 130 West Changjiang Lane, Hefei, Anhui 230036 China; 2 Department of Infectious Diseases and Institute of Clinical Virology, The Second Hospital of Anhui Medical University, No. 678 Furong Lane, Hefei, Anhui 230601 China; 3 MAFLD Research Center, Department of Hepatology, The First Affiliated Hospital of Wenzhou Medical University, No. 2 Fuxue Lane, Wenzhou, 325000 China; 4 Department of Laboratory Medicine, The First Affiliated Hospital of Wenzhou Medical University, Wenzhou, China; 5 Department of Medicine, University of Verona, Verona, Italy; 6 Metabolic Diseases Research Unit, IRCCS Sacro Cuore - Don Calabria Hospital, Negrar Di Valpolicella, Italy; 7 Southampton National Institute for Health and Care Research Biomedical Research Centre, University Hospital Southampton and University of Southampton, Southampton General Hospital, Southampton, UK; 8 Key Laboratory of Diagnosis and Treatment for the Development of Chronic Liver Disease in Zhejiang Province, Wenzhou, China

**Keywords:** Liver fibrosis, Cut-off, Dithiothreitol-oxidizing capacity (DOC), APRI, FIB-4

## Abstract

**Background:**

APRI and FIB-4 scores are used to exclude clinically significant fibrosis (defined as stage ≥ F2) in patients with chronic viral hepatitis. However, the cut-offs for these scores (generated by Youden indices) vary between different patient cohorts. This study aimed to evaluate whether serum dithiothreitol-oxidizing capacity (DOC), i.e., a surrogate test of quiescin sulfhydryl oxidase-1, which is a matrix remodeling enzyme, could be used to non-invasively identify significant fibrosis in patients with various chronic liver diseases (CLDs).

**Methods:**

Diagnostic performance of DOC was compared with APRI and FIB-4 for identifying significant fibrosis. ROC curve analyses were undertaken in: a) two chronic hepatitis B (CHB) cohorts, independently established from hospitals in Wenzhou (*n* = 208) and Hefei (*n* = 120); b) a MASLD cohort from Wenzhou hospital (*n* = 122); and c) a cohort with multiple CLD etiologies (except CHB and MASLD; *n* = 102), which was identified from patients in both hospitals. Cut-offs were calculated using the Youden index. All CLD patients (*n* = 552) were then stratified by age for ROC curve analyses and cut-off calculations.

**Results:**

Stratified by CLD etiology or age, ROC curve analyses consistently showed that the DOC test was superior to APRI and FIB-4 for discriminating between clinically significant fibrosis and no fibrosis, when APRI and FIB-4 showed poor/modest diagnostic performance (*P* < 0.05, *P* < 0.01 and *P* < 0.001 in 3, 1 and 3 cohort comparisons, respectively). Conversely, the DOC test was equivalent to APRI and FIB-4 when all tests showed moderate/adequate diagnostic performances (*P* > 0.05 in 11 cohort comparisons). DOC had a significant advantage over APRI or FIB-4 scores for establishing a uniform cut-off independently of age and CLD etiology (coefficients of variation of DOC, APRI and FIB-4 cut-offs were 1.7%, 22.9% and 47.6% in cohorts stratified by CLD etiology, 2.0%, 26.7% and 29.5% in cohorts stratified by age, respectively). The uniform cut-off was 2.13, yielded from all patients examined. Surprisingly, the uniform cut-off was the same as the DOC upper limit of normal with a specificity of 99%, estimated from 275 healthy control individuals. Hence, the uniform cut-off should possess a high negative predictive value for excluding significant fibrosis in primary care settings. A high DOC cut-off with 97.5% specificity could be used for detecting significant fibrosis (≥ F2) with an acceptable positive predictive value (87.1%).

**Conclusions:**

This proof-of-concept study suggests that the DOC test may efficiently rule out and rule in significant liver fibrosis, thereby reducing the numbers of unnecessary liver biopsies. Moreover, the DOC test may be helpful for clinicians to exclude significant liver fibrosis in the general population.

**Supplementary Information:**

The online version contains supplementary material available at 10.1186/s12916-024-03502-z.

## Background

Chronic liver diseases (CLDs), including metabolic dysfunction-associated steatotic liver disease (MASLD), chronic hepatitis B (CHB), chronic hepatitis C (CHC), alcohol-related liver disease (ALD) and autoimmune hepatitis (AIH), are highly prevalent worldwide [1, 2]. Liver fibrogenesis alters the mechano-signaling properties of the liver, triggers portal hypertension, decreases liver tissue oxygenation, thereby impairing liver regeneration, curtailing liver function, and increasing the odds of chronic kidney disease [3, 4]. If liver fibrosis progresses, it eventually advances to cirrhosis (F4 stage), which annually causes an estimated two million deaths globally [2]. It is desirable not to develop more advanced stages of liver fibrosis and therefore it is important to identify early the initial stages of liver fibrosis. There is an urgent need to identify CLD patients of any etiology at the significant fibrosis stage (F2 stage) to start treatment, such as antiviral therapy in the case of viral hepatitis for halting or reducing the progression rate towards cirrhosis [5]. Hepatic elastometry with different imaging techniques are reliable tools for assessing hepatic fibrosis non-invasively. However, liver biopsy remains the reference method for the assessment of fibrotic stage despite there being inherent problems with liver biopsy, such as potential sampling errors, and low inter-observer agreement and intra-observer reproducibility [6, 7]. In addition, its application is largely restrained due to the invasive nature of liver biopsy with potential post-procedure acute complications [6, 7]. Accurate non-invasive tests (NITs) based on serum as alternatives to liver biopsy examination are in demand. Two simple serum biomarker algorithms, i.e., the aspartate aminotransferase (AST)-to-platelet (PLT) ratio index (APRI) and the fibrosis-4 index (FIB-4), are widely used for the non-invasive assessment of the severity of liver fibrosis in clinical practice, and have been recently incorporated into guidelines by several organizations, including the World Health Organization (WHO) [8–12].

However, the APRI and FIB-4 scores have limitations in their ability to non-invasively assess all stages of liver fibrosis. The major strength of these two simple blood-based scores appears to be the exclusion of significant fibrosis [11–13]. The WHO recommends a strategy combining a low cut-off to rule out the presence of significant fibrosis and a high cut-off to diagnose significant fibrosis (≥ F2) in hepatitis C virus (HCV)-infected or hepatitis B virus (HBV)-infected patients [11, 12]. Normally, a cut-off is calculated based on the Youden index, however, by this approach, highly variable cut-offs of either APRI or FIB-4 scores were documented in various cohorts of CLD patients with the same etiology. Taking APRI as an example, differences of 2.5-fold (0.4–1.0), 3.6-fold (0.235–0.85) and 3.9-fold (0.2–0.77) have been reported in patient cohorts with CHC, CHB and MASLD, respectively (Additional file 1: Table S1). Taking FIB-4 as another example, differences of 2.2-fold (1.0–2.2), 2.0-fold (0.8–1.59) and 3.8-fold (0.46–1.73) have been reported for CHC, CHB and MASLD cohorts, respectively (Additional file 1: Table S2). APRI includes in its equation serum AST and PLT count, whilst FIB-4 includes alanine aminotransferase (ALT), AST and PLT count. These blood parameters are not direct biomarkers of increased fibrogenesis and can be therefore affected by fibrogenesis-unrelated conditions or certain drug interventions, which in essence don’t modify liver fibrosis. In addition, the cut-offs of APRI and FIB-4 scores are affected by age [14–16], and any two cohorts with evidently different age distributions have a high likelihood of giving rise to different cut-offs. As such, the quality of evidence for APRI and FIB-4 scores in assessing liver fibrosis has been rated as low [11, 12]. It is, therefore, plausible to assume that a biomarker that is more pertinent to hepatic fibrogenesis might effectively eliminate the cut-off variability seen with APRI and FIB-4.

Quiescin sulfhydryl oxidase-1 (QSOX1) is a extracellular matrix (ECM) remodeling enzyme [17]. This enzyme is most abundantly expressed in human liver tissue [18] and quantitative proteomics analysis unveiled QSOX1 as the top two plasma proteins among 106 circulating proteins significantly correlated to fibrosis stages in ALD patients [19]. Dithiothreitol-oxidizing capacity (DOC) in human serum is predominantly contributed by QSOX1 [20]. We have recently shown that DOC is a promising biomarker for disease monitoring in HBV-infected patients with normal serum ALT levels [21].

In this proof-of-concept study, we aimed to explore whether the DOC test has potential for efficiently assessing significant liver fibrosis. Our criteria for identifying the DOC test as a novel and reliable, non-invasive biomarker for significant liver fibrosis were as follows. 1) diagnostic performance should not be significantly lower than APRI and FIB-4 scores; and 2) cut-offs calculated by the Youden index in cohorts with the same CLD etiology or different CLD etiologies, and in CLD cohorts of different ages, should be identical; given that diagnostic parameters set for liver biopsy are independent of CLD etiologies and patients’ age. Based on these criteria, two CHB cohorts, a MASLD cohort, and a CLD cohort with multiple CLD etiologies (except CHB or MASLD) were included in this proof-of-concept study.

## Methods

### Participants

From January 2017 to May 2023, a total of 552 patients with CLDs who had undergone liver biopsy were included in the study. Of them, 356 patients were recruited from the First Affiliated Hospital of Wenzhou Medical University, Wenzhou (WZ), China, and 196 patients from the Second Hospital of Anhui Medical University, Hefei (HF), China. The distance between WZ and HF is approximately 700 km. The four cohorts of patients were defined as follows: CHB (WZ) cohort (*n* = 208) -an independent cohort in which all CHB patients were recruited from the hospital in WZ; CHB (HF) cohort (*n* = 120) -an independent cohort in which all CHB patients were recruited from the hospital in HF; MASLD cohort (*n* = 122) -an independent cohort in which all patients including those included in previous studies [22, 23] were recruited from the hospital in WZ; and, finally, a promiscuous CLD cohort (*n*= 102), referred to as ‘other CLD cohort’, in which patients recruited from the two hospitals were merged. The CLD etiologies in this promiscuous cohort included AIH, ALD or CHC but did not contain CHB and MASLD. CHB diagnosis followed the guidelines of American Association for the Study of Liver Diseases (AASLD), 2018 [24]. HBsAg was positive for more than 6 months. MASLD was diagnosed by the presence of hepatic steatosis on liver histology (defined as presence of more than 5% of steatotic hepatocytes) with at least one of the following three coexisting metabolic conditions, i.e., overweight/obesity, type 2 diabetes mellitus, or metabolic dysregulation [25]. Serum samples of 275 healthy individuals were provided by the hospitals affiliated to the Anhui Medical University. This study was approved by the Ethical Committees of the two University hospitals mentioned above (approval No. 2016–246 and 20180347). All study participants provided written informed consent.

### Liver biopsy

Percutaneous liver biopsy was performed using a 16 G needle under ultrasound guidance. The liver biopsy specimens were considered adequate for scoring if they had a length more than 12 mm and contained at least 8 portal tracts. Liver samples were formalin-fixed and paraffin-embedded for the histological analysis. Liver histology was interpreted by two liver pathologists blinded to participants’ clinical characteristics. In cases of discrepancy, the slide was reviewed by another liver pathologist, and the final staging was achieved by consultation amongst three liver pathologists. Liver fibrosis (F0-F4) was staged according to the METAVIR system [26].

### Handling of blood samples

Venous blood samples were collected from all patients and centrifuged to obtain serum samples, which were left at room temperature for 4–8 h and then stored at –80 °C until analysis. Serum DOC activity did not change within 48 h under room temperature. Routine clinical laboratory data within 30 days before or after the liver biopsy were used for calculating APRI, FIB-4 or LiverRisk scores [27].

### DOC activity assay

DOC activity was measured according to our previously validated assay [21] with slight modification. Briefly, serum (15 μL) was diluted with saline (85 μL) and was mixed with 50 μL of the reaction mixture with or without 1 mM dithiothreitol (DTT). The difference of the paired serum tests represented thiol levels in the presence of serum. Saline (100 μL) was mixed with 50 μL of the reaction mixture with or without 1 mM DTT. The difference of the paired saline tests represented thiol levels in the absence of serum. These four reaction mixtures were made for each sample. After a 15-min reaction at 37 °C, the reaction was terminated by adding 200 μL Tris-buffer (200 mM, pH 8.0) containing 6.6 M guanidine hydrochloride and 1 mM 5,5-dithiobis-2-nitrobenzoicacid (DTNB). After 5 min, and within 30 min following initiation of the reaction between DTNB and remaining thiol, absorbance in each reaction (200 μL) was determined at 412 nm using a 96-well plate reader. Serum causing thiol decrease was calculated according to the following formula: [(thiol level in the absence of serum – thiol level in the presence of serum) × 100% ÷ thiol level in the absence of serum]. DOC was expressed as Unit (U)/μL serum. One Unit of DOC was defined as 1% DTT (2% thiol) decrease. Once the thiol decrease exceeded 55%, the serum was diluted for redetermination. The following diagram shows that measuring DOC is quick and easy for laboratories.
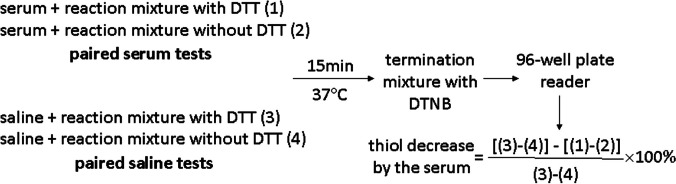


To ensure consistency in batch DOC measurements across different clinical laboratories, we would recommend the following sampling method to maintain a reliable reaction temperature and time in each test. For measuring thiol levels in the presence of serum, serum (15 μL, ice cold) is diluted with saline (85 μL, 37 °C), and is mixed with 50 μL of the reaction mixture with or without 1 mM DTT (37 °C). For measuring thiol levels in the absence of serum, ice cold saline (15 μL) is added to 37 °C saline (85 μL), and then is mixed with 50 μL of the reaction mixture with or without 1 mM DTT (37 °C). During these sampling processes, the test tubes always remain in a 37 °C heat block. After the addition of the first reaction mixture, timer is started and then the reaction mixture is added to subsequent test tube every 10 s. Accordingly, the terminated mixture is added to each test tube every 10 s.

### Statistical analysis

Receiver operating characteristics (ROC) curves were analyzed by SPSS (version 17.0) and MedCalc (version 11.2). Multivariate logistic regression analysis was analyzed by SPSS (version 17.0). Other analyses were performed by GraphPad Prism (version 5.0). Differences between two independent groups were tested with the Mann–Whitney U test. Baseline characteristics of the different CLD cohorts were compared using the χ2 test or the Kruskal–Wallis test. Area under the ROC curve (AUROC) was used to estimate the probability of the correct prediction of liver fibrosis stages. Differences between AUROCs were compared by the DeLong test. Diagnostic accuracy was evaluated by the sensitivity, specificity, positive predictive value (PPV) and negative predictive value (NPV). Optimal cut-offs were selected to maximize the sum of sensitivity and specificity. Coefficient of variation (CoV) was calculated by standard deviation/mean. The Pearson’s correlation coefficient was presented as r. A *P*-value of < 0.05 was considered statistically significant.

## Results

### Characteristics of patient cohorts stratified by CLD etiology

The baseline characteristics of participants included in the four cohorts stratified by CLD etiology are shown in Table 1. The medians of serum DOC, APRI or FIB-4 in the four patient cohorts were used for calculating the corresponding CoVs. The DOC CoV (10%) was markedly lower than the APRI CoV (45%) and the FIB-4 CoV (57%). The implication of such a characteristic of DOC is not yet straightforward. Perhaps, it hints that a less heterogeneous parameter for assessing liver fibrosis may be discovered from DOC.

**Table 1:** Clinical characteristics of the four cohorts of patients stratified by CLD etiology

	**CHB (WZ) cohort (*n* = 208)**	**CHB (HF) cohort (*n* = 120)**	**MASLD cohort (*n* = 122)**	**Other CLD cohort (*n* = 102)**
Male sex, n (%)	164 (78.8)	70 (58.3)	90 (73.8)	31 (30.4)
Age, years	40.0 (32.2, 47.0)	34.0 (29.0, 44.5)	39.5 (31.0, 51.0)	50.0 (43.0, 57.0)
SF, n (%)	73 (35.1)	36 (30)	21 (17.2)	64 (62.7)
DOC, U/μL	2.04 (1.77, 2.50)	2.01 (1.75, 2.28)	1.99 (1.79, 2.31)	2.44 (2.10, 2.99)
APRI	0.46 (0.30, 0.99)	0.38 (0.26, 0.63)	0.47 (0.31, 0.68)	0.93 (0.47, 1.59)
FIB-4	1.12 (0.78, 1.77)	0.86 (0.60, 1.30)	0.82 (0.54, 1.29)	2.38 (1.16, 3.98)

Data of age, DOC, APRI and FIB-4 are presented as medians (interquartile ranges)

*Abbreviations*: *APRI* Aspartate aminotransferase-to-platelet ratio index, *DOC* Dithiothreitol-oxidizing capacity, *FIB-4* Fibrosis-4 index, *SF* Significant fibrosis (histologically defined by ≥ F2 liver fibrosis)

### DOC outperforms or is equivalent to APRI or FIB-4 for diagnosing significant fibrosis

The diagnostic performances of APRI and FIB-4 scores for detecting significant fibrosis in the MASLD cohort were modest with AUROC values of 0.615 and 0.568, respectively. The AUROC value of DOC (0.737) was significantly better than these two values (Table 2). Similar findings were also observed in the CHB (WZ) cohort (Table 2). The performances of APRI and FIB-4 in the other CLD cohorts were moderate with an AUROC value of 0.809 and 0.853, respectively (Table 2). In such case, the AUROC value of DOC (0.798) was not significantly different from these two values (Table 2). Similar results were also found in the CHB (HF) cohort (Table 2). In addition, we performed a multivariable logistic regression analysis between F0-1 patients and ≥ F2 patients, extracted from all CLD patients. The odds ratios of DOC, APRI and FIB-4 were 8.32, 0.80 and 1.41, for detecting ≥ F2 patients, respectively (Additional file 1: Table S3). This also supports the above perception that the diagnostic performance of DOC is in general better than that of APRI and FIB-4.

**Table 2:** Diagnostic performance of DOC, APRI and FIB-4 for significant fibrosis in cohorts of patients stratified by CLD etiology

Etiology	NIT	Cut-off	AUROC	*P* value vs. DOC	Sens (%)	Spec (%)	PPV (%)	NPV (%)
CHB (WZ)	DOC	2.09	0.808 (0.748–0.860)	\	80.8	71.9	60.8	87.4
	APRI	0.59	0.722 (0.656–0.782)	< 0.01	67.1	74.8	59.0	80.8
	FIB-4	1.18	0.640 (0.571–0.705)	< 0.001	60.3	63.0	46.8	74.6
CHB (HF)	DOC	2.15	0.750 (0.656–0.845)	\	61.1	75.0	51.2	81.8
	APRI	0.46	0.722 (0.600–0.844)	> 0.05	54.3	91.6	73.1	82.6
	FIB-4	0.88	0.736 (0.634–0.839)	> 0.05	74.3	65.1	47.3	85.7
MASLD	DOC	2.14	0.737 (0.650–0.812)	\	76.2	69.3	34.0	93.3
	APRI	0.80	0.615 (0.523–0.702)	< 0.05	42.7	86.1	39.1	87.9
	FIB-4	2.63	0.568 (0.475–0.657)	< 0.05	38.1	98.0	80.0	88.4
Other CLDs	DOC	2.18	0.798 (0.707–0.871)	\	85.9	65.8	80.9	73.5
	APRI	0.70	0.809 (0.719–0.881)	> 0.05	79.7	71.1	82.3	67.5
	FIB-4	2.04	0.853 (0.768–0.915)	> 0.05	82.8	89.2	93.0	75.0

*Abbreviations*: *APRI* Aspartate aminotransferase-to-platelet ratio index, *DOC* Dithiothreitol-oxidizing capacity, *FIB-4* fibrosis-4 index, *NIT* Non-invasive test, *NPV* Negative predictive value, *PPV* Positive predictive value, *Sens* Sensitivity, *Spec* Specificity

### Analysis of cut-offs for DOC, APRI and FIB-4

**Fig. 1:**
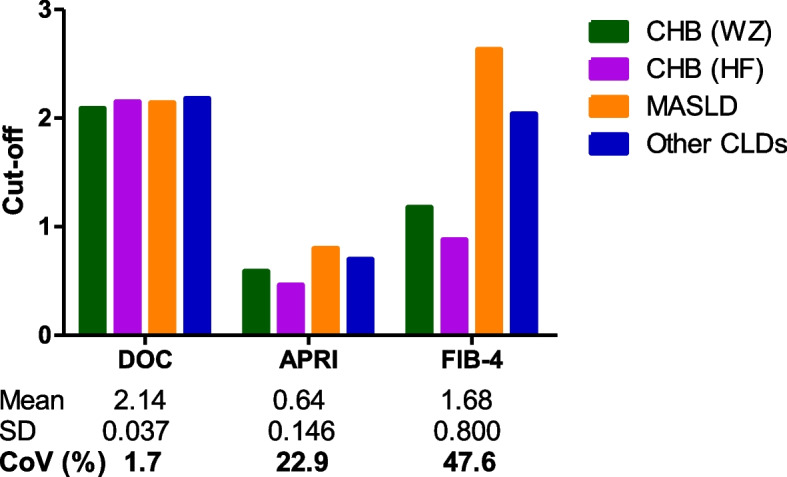
Cut-off variability of DOC, APRI and FIB-4 in patient cohorts stratified by CLD etiology. Abbreviations: SD, standard deviation; CoV, coefficient of variation; DOC, dithiothreitol-oxidizing capacity; APRI, aspartate aminotransferase-to-platelet ratio index; FIB-4, fibrosis-4 index

The optimal cut-offs for DOC, APRI or FIB-4 were obtained by the Youden Index (Table 2). These cut-offs were used to calculate the corresponding CoVs. The CoV of DOC cut-offs across the four cohorts stratified by CLD etiology was 1.7%, whereas the CoVs of APRI or FIB-4 reached as high as 22.9% or 47.6%, respectively (Fig. 1). While the two CHB cohorts had different APRI and FIB-4 cut-offs, these two cohorts had a nearly identical DOC cut-off (Fig. 1). While the MASLD cohort and the other CLD cohort greatly increased FIB-4 cut-offs as compared to the CHB, the DOC cut-offs of the MASLD cohort and the other CLD cohort persistently remained nearly identical to DOC cut-offs of the two CHB cohorts (Fig. 1). Therefore, the DOC cut-off from different cohorts with the same CLD etiology or various etiologies showed a strongly consistent profile.

### Possible factors underlying the uniform DOC cut-off

**Fig. 2:**
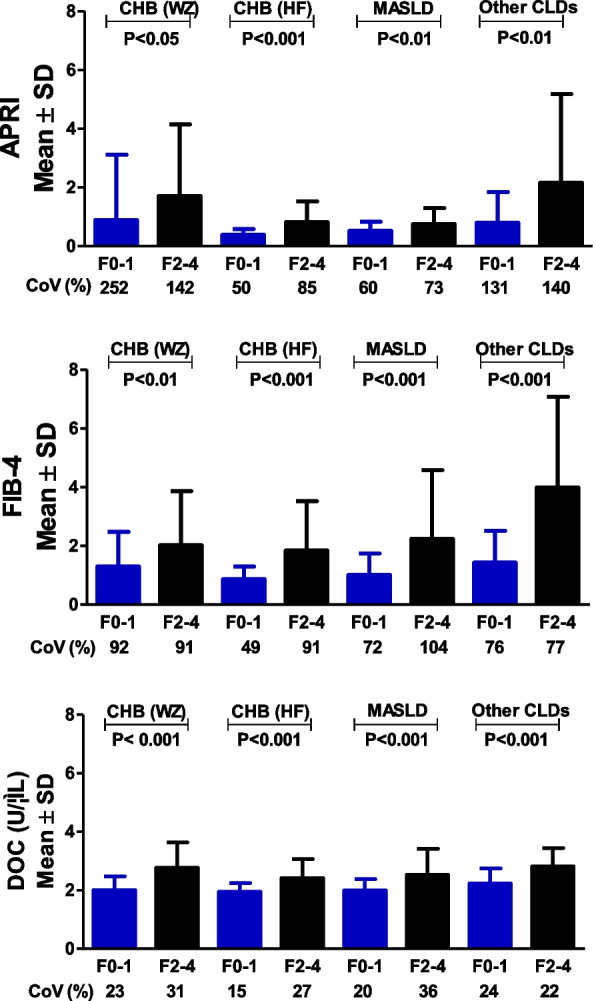
F0-1 and F2-4 variability of APRI, FIB-4 and DOC in patient cohorts stratified by CLD etiology.Abbreviations: SD, standard deviation; CoV, coefficient of variation; DOC, dithiothreitol-oxidizing capacity; APRI, aspartate aminotransferase-to-platelet ratio index; FIB-4, fibrosis-4 index

DOC medians of the four patient cohorts stratified by CLD etiology were less variable compared to those for APRI and FIB-4, as demonstrated above. We then performed data mining to gain insight into multiple variations of DOC, APRI and FIB-4. Regarding APRI and FIB-4, the F0-1 CoV in the four patient cohorts ranged from 50%-252% and 49%-92%, respectively. In contrast, DOC was in a range of 15%-24% (Fig. 2). Regarding APRI and FIB-4, the F2-4 CoV in the four cohorts ranged from 73%-142% and 77%-104%, respectively. In contrast, DOC was in a range of 22%-36% (Fig. 2). Regarding APRI and FIB-4, the CoV of F0-1 mean across the four cohorts was 36% and 22.3%, respectively, whereas the corresponding CoV of DOC was as low as 6.3% (Additional file 1: Fig. S1). Regarding APRI and FIB-4, the CoV of F2-4 mean across the four CLD cohorts was 50.4% and 39.1%, respectively, whereas the corresponding CoV of DOC reached as low as 7.1% (Additional file 1: Fig. S1). In the case of APRI and FIB-4, the CoV of F2-4 mean/F0-1 mean across the four CLD cohorts was 25.5% and 23.1%, respectively, whereas the corresponding CoV of DOC was as low as 2.4% (Additional file 1: Fig. S1). Overall, in all variations examined, DOC had a consistently lower CV than APRI or FIB-4.

### Comparisons between CLD cohorts stratified by age or sex

**Fig. 3:**
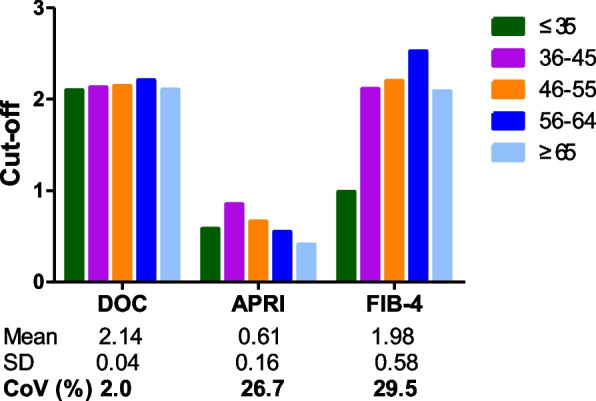
Cut-off variability of DOC, APRI and FIB-4 in pooled CLD cohorts stratified by age. Abbreviations: SD, standard deviation; CoV, coefficient of variation; DOC, dithiothreitol-oxidizing capacity; APRI, aspartate aminotransferase-to-platelet ratio index; FIB-4, fibrosis-4 index

**Fig. 4:**
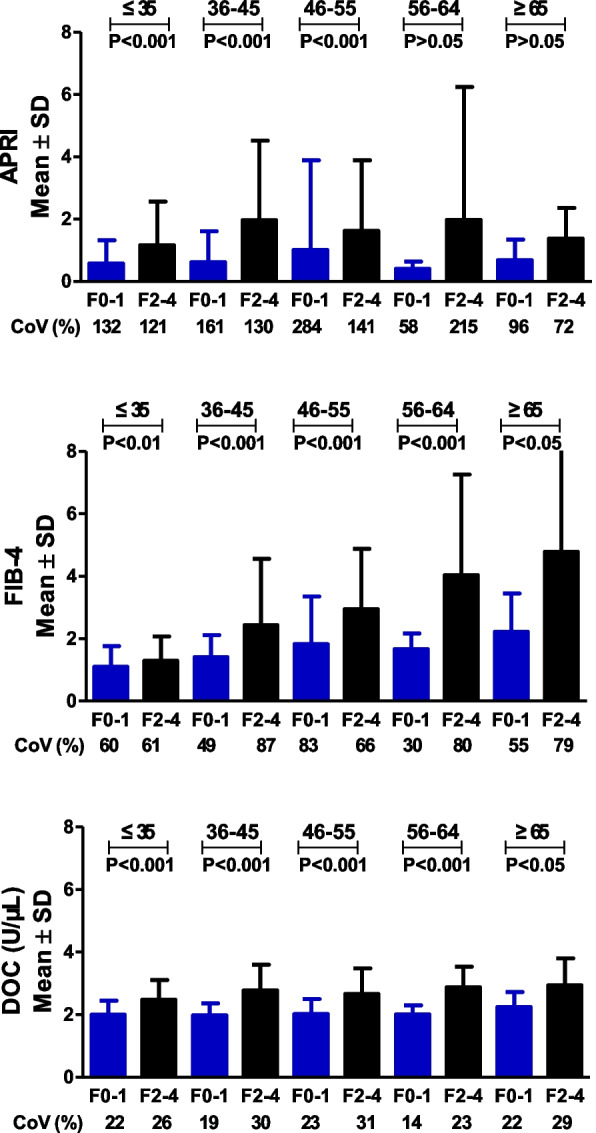
F0-1 and F2-4 variability of APRI, FIB-4 and DOC in pooled CLD cohorts stratified by age. Abbreviations: SD, standard deviation; CoV, coefficient of variation; DOC, dithiothreitol-oxidizing capacity; APRI, aspartate aminotransferase-to-platelet ratio index; FIB-4, fibrosis-4 index

The APRI or FIB-4 cut-off for CHB-associated significant fibrosis needs to be adjusted for age [14, 16]. The FIB-4 cut-off for advanced fibrosis in MASLD also needs to be adjusted for age [15]. We thus examined whether age also influences the DOC cut-off. Patients in the four cohorts stratified by CLD etiology were then regrouped according to an age segmentation model proposed by McPherson et al. [15]. The medians of DOC, APRI or FIB-4 in the patient cohorts stratified by age (Additional file 1: Table S4) were used for calculating the corresponding CoVs. DOC CoV (10%) was lower than APRI CoV (29%) and FIB-4 CoV (36%). Based on AUROC values of the five CLD cohorts stratified by age, DOC was better than or equivalent to APRI or FIB-4 in identifying significant fibrosis (Table 3). Heterogeneity analysis of cut-offs showed that the CoV of DOC cut-offs across the five CLD cohorts was one order of magnitude lower than those of APRI and FIB-4 (Fig. 3). Regarding other variations examined above, including CoV of F0-1 or F2-4 of each cohort (Fig. 4) and CoV of F0-1 mean, F2-4 mean, or F0-1 mean/F2-4 mean across the five CLD cohorts (Additional file 1: Fig. S2), DOC always had a consistently lower CoV than that of APRI or FIB-4. Taken together, age had a marked influence on the cut-offs for APRI or FIB-4 for significant fibrosis diagnosis; in contrast, the influence of age on DOC cut-offs was marginal.

**Table 3:** Diagnostic performance of DOC, APRI and FIB-4 for significant fibrosis in CLD cohorts stratified by age

Age	NIT	Cut-off	AUROC	*P* value vs. DOC	Sens (%)	Spec (%)	PPV (%)	NPV (%)
≤ 35 years	DOC	2.10	0.762 (0.696–0.820)	\	73.5	68.2	43.4	88.6
	APRI	0.58	0.705 (0.635–0.767)	> 0.05	65.3	72.8	44.4	82.3
	FIB-4	0.98	0.582 (0.510–0.652)	< 0.001	59.2	60.5	33.3	81.7
36–45 years	DOC	2.13	0.817 (0.745–0.875)	\	73.3	76.2	56.9	87.0
	APRI	0.85	0.724 (0.645–0.794)	< 0.05	56.8	88.6	67.6	83.0
	FIB-4	2.11	0.640 (0.558–0.717)	< 0.001	43.2	85.7	55.9	78.3
46–55 years	DOC	2.14	0.776 (0.693–0.845)	\	78.7	72.7	72.7	78.7
	APRI	0.66	0.734 (0.649- 0.809)	> 0.05	63.9	80.3	75.0	70.7
	FIB-4	2.20	0.706 (0.693–0.845)	> 0.05	60.7	83.3	77.1	69.6
56–64 years	DOC	2.20	0.931 (0.819–0.984)	\	95.2	77.8	76.9	95.5
	APRI	0.55	0.877 (0.749–0.954)	> 0.05	85.7	85.2	81.8	88.5
	FIB-4	2.52	0.810 (0.670–0.908)	> 0.05	71.4	100.0	100.0	81.8
≥ 65 years	DOC	2.10	0.793 (0.603–0.920)	\	100.0	54.5	78.3	100
	APRI	0.41	0.778 (0.586–0.910)	> 0.05	94.4	63.6	81.0	87.5
	FIB-4	2.10	0.808 (0.620–0.930)	> 0.05	94.4	63.6	81.0	87.5

*Abbreviations: APRI* Aspartate aminotransferase-to-platelet ratio index, *DOC* Dithiothreitol-oxidizing capacity, *FIB-4* Fibrosis-4 index, *NIT* Non-invasive test, *NPV* Negative predictive value, *PPV* Positive predictive value, *Sens* Sensitivity, *Spec* Specificity

It is known that sex may affect diagnostic accuracy, especially in the case of MASLD, which exhibits definite sexual dimorphism [28]. To examine the influence of sex on the DOC cut-off, pooled CLD patients (*n* = 552) were stratified by sex. It was apparent that sex did not significantly affect the DOC cut-off (2.18 for men and 2.14 for women). However, in MASLD patients, the DOC cut-offs were 2.14 and 1.82 for men and women, respectively. Nonetheless, the DOC test was better than APRI and FIB-4 since the differences were 1.41-fold and 4.67-fold for APRI and FIB-4, respectively. It should be noted that the aforementioned 1.17-fold difference between the sexes might be due to a non-uniform representation of men and women in the sample (men = 90 and women = 32). It is possible to hypothesize that a more balanced sex-distribution sample might partly reduce this difference.

### DOC cut-off for ruling-out or ruling-in significant fibrosis

**Fig. 5:**
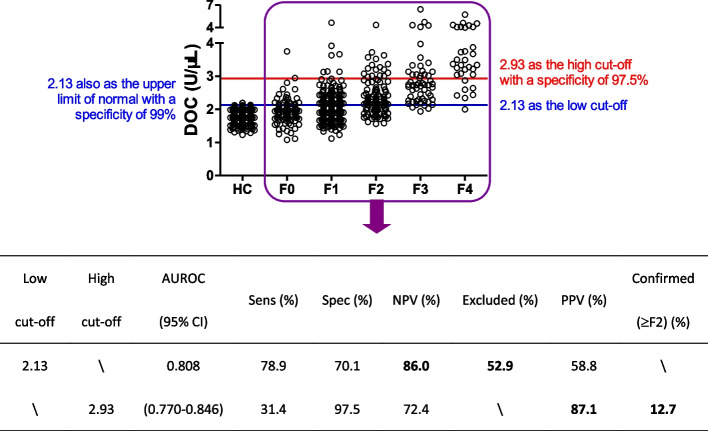
Diagnostic performance of DOC for significant fibrosis. All patients with CLD were combined together for the analysis. Healthy control persons as the HC group were used for assessing the upper limit of normal. Abbreviations: HC, healthy control; Sens, sensitivity; Spec, specificity; PPV, positive predictive value; NPV, negative predictive value; DOC, dithiothreitol-oxidizing capacity

Given the moderate accuracy of APRI and FIB-4 in staging liver fibrosis, to leverage the potential of APRI and FIB-4 in risk stratification, the WHO recommended using a low cut-off for ruling-out significant fibrosis (< F2) and a high cut-off for ruling-in significant fibrosis (≥ F2) [11, 12]. The Youden index-optimized DOC cut-offs yielded from the four CLD cohorts stratified by etiology and the five CLD cohorts stratified by age, were in a narrow range of 2.09–2.18 and 2.10–2.21, respectively (Figs. 1, 3). The Youden index-optimized DOC cut-off yielded from the pooled CLD patient cohort (*n* = 552) was 2.13 (Fig. 5), which fell into the above two ranges. This cut-off generated from a large sample size is recommended as the low cut-off for excluding significant fibrosis. Accordingly, 52.9% CLD patients could be excluded with a NPV of 86.0% in the pooled cohort with a ≥ F2 prevalence of 35% (Fig. 5). Surprisingly, the suggested low cut-off was the same as the DOC upper limit of normal (ULN) with a specificity of 99% (Fig. 5). The ULN was obtained based on 275 healthy persons. Liver function tests of healthy persons and pooled CLD patients are shown in Additional file 1: Table S5. The DOC assay showed an odds ratio prominently higher than the other tests, supporting the motivation to use DOC for identifying ≥ F2 patients. Based on the advantage of DOC it is tempting to assume that the ≥ F2 patients within the pooled CLD patients that included F0-1 patients would gain a even higher odds ratio, thus we compared healthy persons and ≥ F2 patients, indeed, DOC showed an odds ratio much higher than other tests (Additional file 1: Table S6). The ≥ F2 prevalence in primary care settings is likely to be 2.6%-5.0% [2, 29]. Taking the overlap of the ULN and the low cut-off as well as ≥ F2 prevalence in the general population (2.6%-5.0%) into account, the NPV of the low cut-off in primary care settings for ruling-out significant fibrosis could reach as high as 99.5%-98.9%. The high cut-off for predicting significant fibrosis (≥ F2) is suggested to be defined by high specificity [11, 12]. At a specificity of 97.5%, the high cut-off of DOC was 2.93. Accordingly, 12.7% CLD patients were classified into ≥ F2 with a PPV of 87.1% (Fig. 5), which accords with the requirement that a test should be able to correctly classify at least 80% of patients [30]. Overall, the combined application of both low and high cut-offs, liver biopsy could be avoided in roughly 2/3 CLD patients examined in the present study. For CLD patients with a DOC value between the low and high cut-offs, further examination(s) with other NITs, such as enhanced liver fibrosis test and vibration-controlled transient elastography or liver biopsy would be necessary.

### Comparison of DOC and LiverRisk score for diagnosing significant fibrosis

A recent study showed that LiverRisk score predicts long-term liver-related outcomes, such as mortality, hospitalization, and liver cancer in the general population [27]. The LiverRisk score is calculated with an online calculator [31], and is based on age, sex and six routine clinical laboratory parameters, namely, serum levels of ALT, AST, gamma-glutamyltransferase, fasting glucose, total cholesterol, and PLT count. Whether LiverRisk score could be also used to stage significant fibrosis is uncertain. We thus compared its performance with those of DOC, APRI and FIB-4. Of our 552 patients with CLD, almost 60% (i.e. 325 patients) had all the six routine clinical laboratory variables for calculating the LiverRisk score; these patients were then used for the comparison. DOC correlated significantly (*P* < 0.001) to LiverRisk score (*r* = 0.573), APRI (*r* = 0.506) and FIB-4 (*r* = 0.532) (Additional file 1: Fig. S3). AUROC values showed that DOC outperformed each of the LiverRisk score, APRI or FIB-4 in identifying significant fibrosis (Additional file 1: Table S7). The Youden index-optimized DOC cut-off yielded from the 325 patients was 2.13 (Additional file 1: Table S7), which was the same as that yielded from all CLD patients examined (*n* = 552) (Fig. 5).

## Discussion

The main and novel finding of this proof-of-concept study is that the DOC test had better diagnostic performance than the APRI and FIB-4 scores for noninvasively identifying ≥ F2 liver fibrosis. The DOC test had a significant advantage over APRI and FIB-4 scores in showing a consistent diagnostic cut-off across different cohorts of CLD patients. Out of the 10 comparisons between DOC and APRI, the AUROC values showed that DOC was significantly better than APRI in 4 cohorts and was equivalent to APRI in another 6 cohorts (Tables 2, 3 and Additional file 1: Table S7). Out of the 10 comparisons between DOC and FIB-4, the AUROC values showed that DOC was better than FIB-4 in 5 cohorts and was equivalent to FIB-4 in another 5 cohorts (Tables 2, 3 and Additional file 1: Table S7). In general, where APRI or FIB-4 showed only modest diagnostic performances for identifying ≥ F2 liver fibrosis, DOC surpassed APRI and FIB-4 scores in performance. In contrast, when APRI or FIB-4 had moderate/adequate performances for identifying ≥ F2 liver fibrosis, DOC was not inferior to them. The comparisons across different CLD etiologies and age strata clearly show that the DOC test may be a useful biomarker for the non-invasive identification of significant liver fibrosis.

Recently, the APRI and FIB-4 scores have been incorporated into guidelines by several organizations including the WHO for risk stratification of significant liver fibrosis [8–12]. A pitfall of these two blood-based algorithms is their highly variable cut-offs in various patient cohorts with a same CLD etiology (Additional file 1: Tables S1, S2). Consistently, this pitfall was also evident in the present study. In the four CLD cohorts stratified by etiology, the APRI cut-offs showed a 1.74-fold difference in cut-off value between cohorts, with a CoV of 22.9%, whereas the FIB-4 cut-offs showed a 2.99-fold difference in cut-off value between cohorts with a CoV of 47.6%. In contrast, the DOC cut-offs showed only a 1.04-fold difference in cut-off value between cohorts with a CoV of 1.7% (Fig. 1). In the five CLD cohorts stratified by age, for APRI cut-offs there was a 2.44-fold difference in cut-off value between cohorts with a CoV of 26.7%. For FIB-4 cut-offs there was a 2.57-fold difference in cut-off value between cohorts with a CoV of 29.5%. In contrast, for the DOC cut-offs there was only 1.05-fold difference in cut-off value between cohorts with a CoV of 2.0% (Fig. 3). In the case of APRI or FIB-4, the huge variability of these scores with F0-1 and with F2-4 (Figs. 2, 4) and high variations of F0-1 mean, F2-4 mean and F2-4 mean/F0-1 mean (Additional file 1: Figs. S1, 2) may be the underlying factors producing highly variable cut-offs. In the case of DOC, lower variability (Figs. 2, 4) and variations (Additional file 1: Figs. S1, 2) tend to facilitate a more uniform cut-off.

The global burden created by CLDs is considerable [1, 2] and this burden is expected to rise as the epidemics of obesity and type 2 diabetes continue to increase the total numbers of MASLD cases worldwide [32–34]. Significant liver fibrosis may account for about 2.6%-5.0% of the general adult population [2, 29]. Widespread screening for significant fibrosis allows interventions or closer surveillance and since most people are ≥ F2 negative within populations, a NIT that enables clinicians to reliably exclude significant fibrosis is very important to identify people who do not need further tests. By using the low cut-off of DOC, the NPV was 86.0% in the CLD cohort with a ≥ F2 prevalence of 35% (Fig. 5). Since the low cut-off of DOC was the same as the ULN with 99% specificity (Fig. 5), the NPV of DOC thus could reach 99.5%-98.9% in the general population level where ≥ F2 prevalence was 2.6%-5.0%. Thus, DOC test is a highly useful tool to rule out significant liver fibrosis with a particularly high NPV in primary care settings, thus reducing the burden of referrals to secondary care. Recently, the LiverRisk score has been proposed to accurately identify individuals at high risk for future liver-related outcomes in the general population [27]. In our study, DOC significantly correlated to LiverRisk score (Additional file 1: Fig. S3) but outperformed the LiverRisk score in significant fibrosis diagnosis (Additional file 1: Table S7), thus it seems reasonable to compare the performances of DOC and LiverRisk score in predicting the risk of long-term liver-related outcomes in the general population in the future.

The following studies suggest a link between DOC and hepatic fibrogenesis. The activity of human serum DOC is contributed mainly by QSOX1 [20]. QSOX1 is a secreted protein [17]. Extracellular QSOX1 controls ECM composition by catalyzing cysteine cross-linking or disulfide bond formation [17]. QSOX1 inhibition strongly disrupts incorporation of laminin, a key basement membrane component, into ECM. The resultant ECM is more elastic as evidenced by atomic force microscopy [17]. QSOX1 Inhibition also leads to reduced deposition of two additional major ECM components (fibronectin and collagen) in ECM [35, 36]. Of QSOX1-facilitated matrix remodeling events, QSOX1 preferentially adheres to fibronectin, laminins and Matrigel relative to collagen and gelatin, through the formation of mixed disulfides between QSOX1 and cysteine-rich ECM proteins [37]. The role of secreted QSOX1 in ECM is an emerging and impactful concept without receiving adequate attention it deserves in the field of liver fibrosis research, however, it should be emphasized that QSOX1 mRNA is most abundantly expressed in the liver among various human tissues, including the heart, brain, placenta, lung, skeletal muscle, kidney and pancreas [18]. Two outstanding studies have revealed an association between QSOX1 and liver fibrosis. Niu et al. found that QSOX1 ranks in the top two plasma proteins among 106 circulating proteins significantly correlated to fibrosis stages in ALD patients [19]. Baker et al. showed that fast recurrence of liver fibrosis in HCV-infected patients post liver transplantation is associated with a marked elevation of serum QSOX1 protein [38].

The optimal tool for diagnosing liver fibrosis should be [39] i) related to ECM deposition, ii) useable across different etiologies of CLD, iii) easy to perform, cost-effective, readily available, and reproducible across diagnostic platforms, and iv) resistant to the influence of physiologic variations such as age and sex. The DOC test is associated with ECM deposition and has a uniform cut-off across different CLD etiologies. The DOC test is a common colorimetric analysis without employing expensive chemicals and instruments, and it can be standardized across diagnostic platforms given the simple test procedure. Thus, the DOC test could be considered as an ideal NIT implemented in clinical laboratories of primary, secondary, and tertiary healthcare centers, and used by physicians or healthcare payers to triage CLD patients for treatment options and proactive disease management.

Whether QSOX1 plays a role in elevating oxidative stress during the progression of CLDs remains to be fully determined. Nrf2 is a master antioxidant transcription factor. QSOX1 may impair Nrf2 activation in hepatocellular carcinoma cells [40], suggesting that the over-expression of hepatic QSOX1 may promote oxidative stress. QSOX1 was initially found to be a specific biomarker for indentifying acute decompensated heart failure [41], while more recently it was revealed to be a biomarker for CLDs [19, 21]. Contrary to the trajectory, serum gamma glutamyl transferase concentration, a non-specific liver test, has evolved to be a biomarker of cardiometabolic health [42]. We have found that the DOC test could not be used for indentifying acute myocardial infarction [21]. In addition, QSOX1 is only sensitive in the case of acute decompensated heart failure, but not stable chronic heart failure [41]. Therefore, QSOX1 or DOC seems to have little potential in cardiovascular risk assessment and stratification in patients with CLDs.

The current proof-of-concept study has some important limitations. Independent cohorts of alcohol-related liver disease, hepatitis C, and autoimmune liver disease were lacking; rather the relevant patients due to the small numbers were included in a cohort referred as other CLDs, namely the promiscuous CLD cohort. In addition, all CLD patients were of Chinese ethnicity. Future larger studies including patients with various CLD etiologies from different countries are needed. In addition, prospective studies are also needed to investigate the relationships between the DOC test and fibrosis development or prognosis following drug treatments.

## Conclusions

We report the diagnostic performance of DOC for non-invasively identifying significant liver fibrosis in several cohorts of CLD patients stratified by etiology or age. The DOC test is simple and cost-effective and could therefore be easily applied to routine clinical practice. The DOC test is accurate for ruling-out and ruling-in significant fibrosis, obviating the need for unnecessary liver biopsies. The DOC test may also be helpful for clinicians to rule out significant fibrosis at the population level. We suggest that future studies based on a variety of CLD etiologies and larger sample cohorts of different ethnicities are needed to further corroborate our findings.

### Supplementary Information

Additional file 1: Table S1. Examples of different APRI cut-offs for the non-invasive identification of significant (≥F2) liver fibrosis in patients with CLD of different etiologies. Table S2. Examples of different FIB-4 cut-offs for the non-invasive identification of significant (≥F2) liver fibrosis in patients with CLD of different etiologies. Table S3. Comparison of DOC, APRI and FIB-4 between F0-1 and F2-4 in all CLD patients. Table S4. Clinical characteristics of pooled CLD cohorts stratified by age. Table S5. Comparison of clinical characteristics between healthy controls (HC) and all CLD patients. Table S6. Comparison of clinical characteristics between healthy controls (HC) and patients with significant (≥F2) liver fibrosis. Table S7. Comparison between DOC and LiverRisk, APRI or FIB-4 diagnostic performance for staging significant (≥F2) liver fibrosis in CLD patients. Fig. S1. F0-1 mean, F2-4 mean and F2-4 mean/F0-1 mean of DOC, APRI and FIB-4 in patient cohorts stratified by CLD etiology and the corresponding variability between cohorts. Fig. S2. F0-1 mean, F2-4 mean and F2-4 mean/F0-1 mean of DOC, APRI and FIB-4 in pooled CLD cohorts stratified by age and the corresponding variability between cohorts. Fig. S3. Univariable linear correlations of DOC with LiverRisk, APRI or FIB-4 scores.

## Data Availability

All data generated or analyzed during this study are included in this article and its supplementary material files. Further enquiries can be directed to the corresponding authors.

## Abbreviations

AIH: Autoimmune hepatitis
ALD: Alcohol-related liver disease
ALT: Alanine aminotransferase
APRI: Aspartate aminotransferase-to-platelet ratio index
AST: Aspartate aminotransferase
AUROC: Area under the ROC curve
CHB: Chronic hepatitis B
CHC: Chronic hepatitis C
CLDs: Chronic liver diseases
CoV: Coefficient of variation
DOC: Dithiothreitol-oxidizing capacity
DTNB: 5,5-Dithiobis-2-nitrobenzoicacid
DTT: Dithiothreitol
ECM: Extracellular matrix
FIB-4: Fibrosis-4 index
HF: Hefei
MASLD: Metabolic dysfunction-associated steatotic liver disease
NITs: Non-invasive tests
NPV: Negative predictive value
PPV: Positive predictive value
QSOX1: Quiescin sulfhydryl oxidase 1
ROC: Receiver operating characteristics
Sens: Sensitivity
Spec: Specificity
ULN: Upper limit of normal
WHO: World health organization
WZ: Wenzhou
