# Supplementary material for: Serum dithiothreitol-oxidizing capacity (DOC) is a promising biomarker for excluding significant liver fibrosis: a proof-of-concept study
Source: BMC Med. 2024 Jul 2;22:278. doi: 10.1186/s12916-024-03502-z (PMC11221035; doi:10.1186/s12916-024-03502-z)
Supplement: Supplementary file 1 — Additional file 1: Table S1. Examples of different APRI cut-offs for the non-invasive identification of significant (≥F2) liver fibrosis in patients with CLD of different etiologies. Table S2. Examples of different FIB-4 cut-offs for the non-invasive identification of significant (≥F2) liver fibrosis in patients with CLD of different etiologies. Table S3. Comparison of DOC, APRI and FIB-4 between F0-1 and F2-4 in all CLD patients. Table S4. Clinical characteristics of pooled CLD cohorts stratified by age. Table S5. Comparison of clinical characteristics between healthy controls (HC) and all CLD patients. Table S6. Comparison of clinical characteristics between healthy controls (HC) and patients with significant (≥F2) liver fibrosis. Table S7. Comparison between DOC and LiverRisk, APRI or FIB-4 diagnostic performance for staging significant (≥F2) liver fibrosis in CLD patients. Fig. S1. F0-1 mean, F2-4 mean and F2-4 mean/F0-1 mean of DOC, APRI and FIB-4 in patient cohorts stratified by CLD etiology and the corresponding variability between cohorts. Fig. S2. F0-1 mean, F2-4 mean and F2-4 mean/F0-1 mean of DOC, APRI and FIB-4 in pooled CLD cohorts stratified by age and the corresponding variability between cohorts. Fig. S3. Univariable linear correlations of DOC with LiverRisk, APRI or FIB-4 scores. [file 12916_2024_3502_MOESM1_ESM.doc]

**Table S1.** **Examples of different APRI cut-offs for the non-invasive identification of significant (≥F2) liver fibrosis in patients with CLD of different etiologies**.

| **CLD etiology** | **Cut-off(s)** | **Number of studies (number of patients)** | **Reference (#PMID)** |
| --- | --- | --- | --- |
| CHC | 0.4 | 5 (836) | Hepatology. 2011;53:726-736 (21319189) |
|  | <0.5 | 11 (2052) |  |
|  | 0.6 | 3 (531) |  |
|  | 0.7 | 4 (609) |  |
|  | 1 | 3 (821) |  |
|  | 0.6-1.0 | 13 (2424) |  |
| CHB | 0.235-0.425 | 4 (1260) | Hepatology. 2015;61:292-302 (25132233) |
|  | 0.535-0.85 | 5 (906) |  |
| MASLD | 0.2 | 1 (101) | Obes Surg. 2017;27:115-125 (27220852) |
|  | 0.4 | 1 (73) | Obes Surg. 2020;30:1249-1257 (31953745) |
|  | 0.43 | 1 (242) | J Gastroenterol Hepatol. 2011;26:1536-1543 (21950746) |
|  | 0.43 | 1 (373) | Front Endocrinol (Lausanne). 2023;14:1090598 (36793287) |
|  | 0.45 | 1 (100) | BMJ Open Gastroenterol. 2019;6:e000288 (31275584) |
|  | 0.6 | 1 (207) | United European Gastroenterol J. 2019;7:1124-1134 (31662869) |
|  | 0.70 | 1 (251) | Front Med (Lausanne). 2022;9:869190 (35492369) |
|  | 0.77 | 1 (52) | J Gastroenterol. 2008;43:720-728 (18807134) |

**Table S2.** **Examples of different FIB-4 cut-offs for the non-invasive identification of significant (≥F2) liver fibrosis in patients with CLD of different etiologies**.

| **CLD etiology** | **Cut-off(s)** | **Number of studies (number of patients)** | **Reference (#PMID)** |
| --- | --- | --- | --- |
| CHC | 1.0 | 1 (830) | Hepatology. 2006;43:1317-1325. (16729309) |
|  | 1.0 | 1 (100) | Hepatol Res. 2015;45:560-570. (24995544) |
|  | 1.17 | 1 (100) | J Med Virol. 2020. (32558950) |
|  | 1.505 | 1 (208) | Cells. 2019;8:1003. (31470644) |
|  | 1.86 | 1 (107) | Clin Chim Acta. 2008;397:51-54. (18692034) |
|  | 1.86 | 1 (138) | Biomed Res Int. 2019;2019:2639248. (31061822) |
|  | 2.2 | 1 (110) | Hepatol Res. 2016;46:752-757. (26583748) |
| CHB | 0.4 | 1 (390) | World J Gastroenterol. 2017;23:7425-7432. (29151696) |
|  | 0.8-1.1 | 5 (1026) | Hepatology. 2015;61:292-302. (25132233) |
|  | 0.96 | 1 (179) | Med Mal Infect. 2019;49:607-615. (30871816) |
|  | 1.38 | 1 (126) | J Magn Reson Imaging. 2017;45:1186-1194. (27563840) |
|  | 1.59 | 1 (319) | J Viral Hepat. 2014;21:917-920. (25131445) |
| MASLD | 0.46 | 1 (373) | Front Endocrinol (Lausanne). 2023;14:1090598. (36793287) |
|  | 0.66 | 1 (73) | Obes Surg. 2020;30:1249-1257. (31953745) |
|  | 0.74 | 1 (101) | Obes Surg. 2017;27:115-125. (27220852) |
|  | 0.89 | 1 (207) | United European Gastroenterol J. 2019;7:1124-1134. (31662869) |
|  | 1.45 | 1 (242) | J Gastroenterol Hepatol. 2011;26:1536-1543. (21950746) |
|  | 1.73 | 1 (251) | Front Med (Lausanne). 2022;9:869190. (35492369) |

**Table S3. Comparison of DOC, APRI and FIB-4 between F0-1 and F2-4 in all CLD patients.**

| **NIT** | **F0-1 (n=358)** | **F2-4 (n=194)** | ***P* value** | **Odds ratio (95% CI)** | ***P* value** |
| --- | --- | --- | --- | --- | --- |
| DOC, U/μL | 1.95 (1.74, 2.19) | 2.48 (2.17, 3.07) | <0.0001 | 8.32 (4.96-13.94) | <0.0001 |
| APRI | 0.39 (0.28, 0.60) | 0.89 (0.50, 1.60) | <0.0001 | 0.80 (0.66-0.97) | 0.023 |
| FIB-4 | 1.23 (0.73, 1.90) | 2.13 (1.12, 3.40) | <0.0001 | 1.41 (1.09-1.82)  56.34 | 0.009 |

Note: Data are presented as medians (inter-quartiles). *P* values based on the Mann-Whitney U test for quantitative data with non-normal distribution. Multivariable logistic regression models (Odds ratios and corresponding 95%CIs) were adjusted for sex and age (reference group F0-1).

*Abbreviations:* APRI, aspartate aminotransferase-to-platelet ratio index; DOC, dithiothreitol-oxidizing capacity; FIB-4, fibrosis-4 index; NIT, non-invasive test.

**Table S4. Clinical characteristics of pooled CLD cohorts stratified by age.**

|  | **≤35 years**  **(n=198)** | **36-45 years**  **(n=150)** | **46-55 years**  **(n=127)** | **56-64 years**  **(n=48)** | **≥65 years**  **(n=29)** | ***P*-value** |
| --- | --- | --- | --- | --- | --- | --- |
| Male sex, n (%) | 147 (74.2) | 106 (70.7) | 66 (52.0) | 29 (60.4) | 7 (24.1) | <0.001 |
| Age, years | 30.0 (25.5, 30.0) | 40.0 (38.0, 43.0) | 50.0 (47.0, 53.0) | 59.0 (57.0, 62.0) | 67.0 (66.0, 70.0) | <0.001 |
| SF, n (%) | 49 (24.7) | 45 (30) | 61 (48.0) | 21 (43.7) | 18 (62.1) | <0.001 |
| DOC, U/μL | 2.01 (1.78, 2.30) | 2.00 (1.80, 2.43) | 2.18 (1.82, 2.66) | 2.25 (1.96, 2.74) | 2.56 (2.19, 3.02) | <0.001 |
| APRI | 0.44 (0.28, 0.70) | 0.47 (0.32, 0.87) | 0.55 (0.35, 1.17) | 0.51 (0.30, 0.99) | 0.85 (0.39, 1.41) | <0.01 |
| FIB-4 | 0.87 (0.61, 1.72) | 1.44 (0.83, 2.04) | 1.78 (1.28, 2.59) | 1.95 (1.54, 2.74) | 2.56 (2.19, 3.02) | <0.001 |

Note: Data for age, DOC, APRI and FIB-4 are presented as medians (inter-quartiles). The clinical and biochemistry parameters across the five age groups were compared using the Kruskal‐Wallis test for continuous variables and χ2 test for categorical variables.

*Abbreviations:* APRI, aspartate aminotransferase-to-platelet ratio index; DOC, dithiothreitol-oxidizing capacity; FIB-4, fibrosis-4 index; SF, significant fibrosis (histologically defined by ≥F2 liver fibrosis).

**Table S5.** **Comparison of clinical characteristics between healthy controls (HC) and all CLD patients.**

|  | **HC (n=275)** | **CLD (n=552)** | ***P* value** | **Odds ratio (95% CI)** | ***P* value** |
| --- | --- | --- | --- | --- | --- |
| Male sex, n (%) | 185 (67.3) | 355 (64.3) | 0.399 | \ | \ |
| Age, years | 41.0 (30.0, 53.0) | 40.0 (32.0, 50.0) | 0.871 | \ | \ |
| DOC, U/μL | 1.77 (1.62, 1.91) | 2.10 (1.80, 2.40) | <0.0001 | 16.66 (4.93-56.34) | <0.0001 |
| ALT, U/L | 19.0 (14.0, 26.0) | 49.0(30.0, 94.2) | <0.0001 | 1.10 (1.06-1.14) | <0.0001 |
| AST, U/L | 19.0 (16.0, 23.0) | 37.0 (26.0, 63.2) | <0.0001 | 1.07 (1.01-1.13) | 0.022 |
| TB, μmol/L | 13.0 (10.0, 17.0) | 13.0 (9.9, 18.0) | 0.741 | 1.11 (1.05-1.16) | <0.0001 |
| DB, μmol/L | 4.10 (2.60, 5.60) | 4.00 (3.00, 6.00) | 0.282 | 0.83 (0.76-0.92) | <0.0001 |
| ALB, g/L | 46.3 (42.9, 48.8) | 37.8 (9.0, 43.5) | <0.0001 | 0.74 (0.69-0.80) | <0.0001 |

Note: Data are presented as proportions, medians (inter-quartiles) according to the original data distribution. *P* values based on the Mann-Whitney U test for quantitative data with non-normal distribution, and the chi-square test for qualitative data. Multivariable logistic regression models (Odds ratios and corresponding 95%CIs) were adjusted for sex and age (reference group HC).

*Abbreviations:* DOC, dithiothreitol-oxidizing capacity; ALT, alanine aminotransferase; AST, aspartate aminotransferase; TB, total bilirubin; DB, direct bilirubin; ALB, albumin.

**Table S6.** **Comparison of clinical characteristics between healthy controls (HC) and patients with significant (≥F2) liver fibrosis.**

|  | **HC (n=275)** | **F2-4 (n=194)** | ***P* value** | **Odds ratio (95% CI)** | ***P* value** |
| --- | --- | --- | --- | --- | --- |
| Male sex, n (%) | 185 (67.3) | 109 (56.2) | 0.014 | \ | \ |
| Age, years | 41.0 (30.0, 53.0) | 46.0 (35.0, 54.0) | 0.008 | \ | \ |
| DOC, U/μL | 1.77 (1.62, 1.91) | 2.48 (2.17, 3.07) | <0.0001 | 10751 (216-534538) | <0.0001 |
| ALT, U/L | 19.0 (14.0, 26.0) | 72.0 (39.0, 139.5) | <0.0001 | 1.06 (0.99-1.15) | 0.084 |
| AST, U/L | 19.0 (16.0, 23.0) | 55.0 (35.0, 94.0) | <0.0001 | 1.15 (1.00-1.31) | 0.046 |
| TB, μmol/L | 13.0 (10.0, 17.0) | 15.2 (12.0, 23.7) | <0.0001 | 1.22 (1.05-1.42) | 0.008 |
| DB, μmol/L | 4.10 (2.60, 5.60) | 5.00 (3.00, 9.00) | <0.0001 | 0.71 (0.56-0.89) | 0.003 |
| ALB, g/L | 46.3 (42.9, 48.8) | 35.4 (10.0, 41.0) | <0.0001 | 0.65 (0.52-0.82) | <0.0001 |

Note: Data are presented as proportions, medians (inter-quartiles) according to the original data distribution. *P* values based on the Mann-Whitney U test for quantitative data with non-normal distribution, and the chi-square test for qualitative data. Multivariable logistic regression models (Odds ratios and corresponding 95%CIs) were adjusted for sex and age (reference group HC).

*Abbreviations:* DOC, dithiothreitol-oxidizing capacity; ALT, alanine aminotransferase; AST, aspartate aminotransferase; TB, total bilirubin; DB, direct bilirubin; ALB, albumin.

**Table S7. Comparison between DOC and LiverRisk, APRI or FIB-4 diagnostic performance for staging** **significant (≥F2) liver fibrosis in CLD patients.**

| **NIT** | **Cut-off** | **AUROC** | ***P*-value**  **vs. DOC** | **Sens (%)** | **Spec (%)** | **PPV (%)** | **NPV (%)** |
| --- | --- | --- | --- | --- | --- | --- | --- |
| DOC, U/μL | 2.13 | 0.790 (0.742-0.833) | \ | 76.9 | 71.4 | 51.1 | 88.8 |
| LiverRisk, kPa | 6.50 | 0.666 (0.612-0.718) | < 0.001 | 67.0 | 64.1 | 42.1 | 83.3 |
| APRI | 0.59 | 0.700 (0.647-0.749) | < 0.01 | 64.8 | 73.5 | 48.8 | 84.3 |
| FIB-4 | 1.74 | 0.647 (0.593-0.699) | < 0.001 | 39.6 | 85.5 | 51.4 | 78.4 |

Note: A total of 325 CLD patients (203 from CHB (WZ) *plus* 122 from MASLD) with LiverRisk scores available were used for the analyses.

*Abbreviations*: APRI, aspartate aminotransferase-to-platelet ratio index; DOC, dithiothreitol-oxidizing capacity; FIB-4, fibrosis-4 index; NIT, non-invasive test; NPV, negative predictive value; PPV, positive predictive value; Sens, sensitivity; Spec, specificity.

**Fig. S1. F0-1 mean, F2-4 mean and F2-4 mean/F0-1 mean of DOC, APRI and FIB-4 in patient cohorts stratified by CLD etiology and the corresponding variability between cohorts.**

*Abbreviations*: APRI, aspartate aminotransferase-to-platelet ratio index; CoV, coefficient of variation; DOC, dithiothreitol-oxidizing capacity; FIB-4, fibrosis-4 index.

**Fig. S2.** **F0-1 mean, F2-4 mean and F2-4 mean/F0-1 mean of DOC, APRI and FIB-4 in pooled CLD cohorts stratified by age and the corresponding variability between cohorts.**

*Abbreviations*: APRI, aspartate aminotransferase-to-platelet ratio index; CoV, coefficient of variation; DOC, dithiothreitol-oxidizing capacity; FIB-4, fibrosis-4 index.

**Fig. S3. Univariable linear correlations of DOC with LiverRisk, APRI or FIB-4** **scores.** A total of 325 CLD patients (203 from CHB (WZ) plus 122 from MASLD) with LiverRisk scores available were used for the analysis. All CLD patients (n=552) with FIB-4 and APRI data were used for the analysis.

*Abbreviations*: APRI, aspartate aminotransferase-to-platelet ratio index; DOC, dithiothreitol-oxidizing capacity; FIB-4, fibrosis-4 index.
